# Transition in the growth mode of plasmonic bubbles in binary liquids†

**DOI:** 10.1039/d2sm00315e

**Published:** 2022-05-12

**Authors:** Marvin Detert, Yibo Chen, Harold J. W. Zandvliet, Detlef Lohse

**Affiliations:** Physics of Fluids Group, Department of Science and Technology, Max Planck Center for Complex Fluid Dynamics and J. M. Burgers Centre for Fluid Dynamics, MESA+ Institute for Nanotechnology, University of Twente 7500AE Enschede The Netherlands d.lohse@utwente.nl; Physics of Interfaces and Nanomaterials, MESA+ Institute for Nanotechnology, University of Twente 7500 AE Enschede The Netherlands; Laboratory for Fluid Physics, Pattern Formation and Biocomplexity, Max Planck Institute for Dynamics and Self-Organization 37077 Göttingen Germany

## Abstract

Multi-component fluids with phase transitions show a plethora of fascinating phenomena with rich physics. Here we report on a transition in the growth mode of plasmonic bubbles in binary liquids. By employing high-speed imaging we reveal that the transition is from slow evaporative to fast convective growth and accompanied by a sudden increase in radius. The transition occurs as the three-phase contact line reaches the spinodal temperature of the more volatile component leading to massive, selective evaporation. This creates a strong solutal Marangoni flow along the bubble which marks the beginning of convective growth. We support this interpretation by simulations. After the transition the bubble starts to oscillate in position and in shape. Though different in magnitude the frequencies of both oscillations follow the same power law 
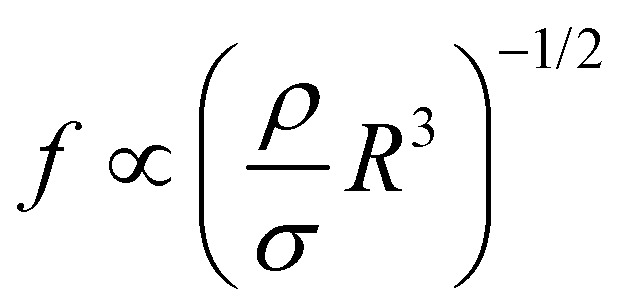
, which is characteristic of bubble shape oscillations, with the surface tension *σ* as the restoring force and the bubble's added mass as inertia. The transitions and the oscillations both induce a strong motion in the surrounding liquid, opening doors for various applications where local mixing is beneficial.

## Abstract 2

Observation and explanation of a transition in the growth mode of plasmonic microbubbles in binary liquids.

## Graphical abstract

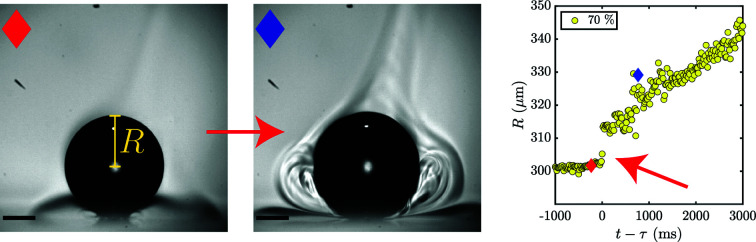

## 1 Introduction

Bubbles and bubble nucleation are omnipresent in a wide variety of phenomena in nature and technology ranging from everyday life, such as boiling and sparkling wine, via industrially relevant fields like catalysis and electrolysis, to medical applications, such as ultrasound contrast agents.^1–4^ The richness of phenomena becomes even larger in multicomponent systems,^5^ in particular when they involve phase transitions.^6^

One example of such a system is a plasmonic bubble, which is created at immersed noble metal nanoparticles irradiated by a laser.^7–13^ In such a system light absorption via surface plasmon resonance leads to abundant heat causing the nucleation and growth of bubbles.^1,6^ These bubbles are initially almost solely composed of vapor, but eventually grow to be dominated by diffusion of dissolved gas from the surrounding liquid.^14–17^ Multiple recent studies elucidated a variety of fascinating phenomena occurring with plasmonic bubbles, such as a competition between solutal and thermal Marangoni forces leading to a bouncing bubble in the early growth stages,^18^ the formation of droplet plumes due to a Marangoni instability in ternary liquids^19^ or the deposition of gold nanoparticles from a suspension.^20^ Moreover, a multitude of potential applications of these bubbles has been demonstrated, *e.g.* in catalysis,^21–23^ medicine^24–28^ or microfluidics.^29–32^

This paper adds another surprising phenomenon to the rich phenomenology of physiochemical hydrodymanics involving plasmonic bubbles in binary liquids. Again, it builds on the preferential evaporation of one component of the binary liquid, which can lead to a bouncing bubble in the early growth stage via competing thermal and solutal Marangoni forces.^18^ The key idea here is to carry this on even further and to study what happens if we now take a drastic difference in the volatility of the two components of the binary liquids, much larger than we did before in Zeng *et al.* (2021).^18^ As we will see, this will lead to even stronger preferential evaporation and thereby larger concentration gradients, which gives rise to two new phenomena: one is a sudden, instantaneous transition in growth mode that is found for a variety of binary liquids. We study this experimentally via highspeed imaging and compare our results with simulations in order to identify the driving mechanism. The other phenomenon is a bubble oscillation right after the transition with a frequency that differs by an order of magnitude from that of the bouncing bubble. To rationalize our findings we propose a mechanism based on the competition between inertia and surface tension.

The paper is organised as follows: first, the experimental setup and the preparation of binary liquids are introduced in Section 2. Then, the transition in the growth mode is described in Section 3.1, followed by an detailed examination in Section 3.2. From this a mechanism is derived in Section 3.3 and supported by simulations. In Section 4 the bubble oscillations are discussed and compared to established theoretical predictions. Finally, the paper ends with conclusions and an outlook on further experiments and applications in Section 5.

## 2 Experimental setup and procedure

### 2.1 Experimental setup

A fused silica substrate decorated with an array of gold nanoparticles (*d* ≈ 70 nm) was placed in a quartz cuvette (10 × 10 × 45 mm) filled with a binary liquid (as described in detail in an earlier study^19^). To create plasmonic bubbles the array was irradiated with a continuous, 532 nm laser (Cobalt Samba, 532 nm, 300 mW) from the bottom with a laser spot diameter of *d*_L_ ≈ 40 μm. A polarizer and a half-wave plate were used to control the laser power which was measured via a photodiode power sensor (S130C; ThorLabs). The bubble dynamics was captured with two high-speed cameras; one for the bottom-view (Photron SA7/NOVA S16) and the other for the side-view (Photron SAZ/NOVA S12). Both cameras were equipped with a 5× working distance objective (LMPLFLN; Olympus) and were operated at frame rates up to 5 kHz. Two light sources, a Schott ACE I and a Schott KL2500, provided back light illumination for the high-speed imaging.

### 2.2 Binary liquid preparation

In order to study a wide range of parameters multiple binary liquids at different mixing ratios were prepared. We first performed a coarse scan of the binary liquids in steps of 10% volume fraction. Then, regions of interest were mapped with steps of 2% volume fraction. To avoid evaporation, the mixtures were immediately transferred to the cuvette after preparation. Every binary liquid we used was composed of acetone with another miscible organic solvent, such as benzyl alcohol (BnOH), cyclohexanone (CHN), 1-heptanol and 1-butanol all from Sigma-Aldrich. These liquids were chosen as they have a significantly higher boiling temperature than acetone (see Table 1) and hence are less volatile. Additionally, they have a higher refractive index than acetone, in particular BnOH. Therefore, changing the local concentration due to preferential evaporation will change the refractive index, which should become qualitatively visible in the imaging.

**Table 1:** Properties of the pure liquids. All values are given at ambient pressure and room temperature, the latent heat of vaporization is at the respective boiling temperature^33,34^

Property	Benzyl alcohol	Cyclohexanone	1-Heptanol	1-Butanol	Acetone
Refractive index	1.54	1.45	1.42	1.40	1.36
Boiling temperature (K)	478.5	428.5	449.7	390.8	329.2
Density (kg m^−3^)	1046	947	822	810	790
Thermal cond. (W (m K)^−1^)	0.156	0.149	0.159	0.135	0.157
Heat capacity (J (mol K)^−1^)	124.8	124.8	173.5	173.7	123.7
Latent heat of vap. (kJ mol^−1^)	46.9	37.3	46.0	43.3	29.1
Surface tension (mN m^−1^)	39.1	34.9	26.8	24.5	23.7
Viscosity (mPa s)	5.93	2.26	6.92	2.94	0.32
Vapor pressure (kPa)	0.008	0.40	0.02	0.64	24.50
Transition	Yes	Yes	Yes	No	—

## 3 Transition in growth mode

### 3.1 Phenomenology

**Fig. 1:**
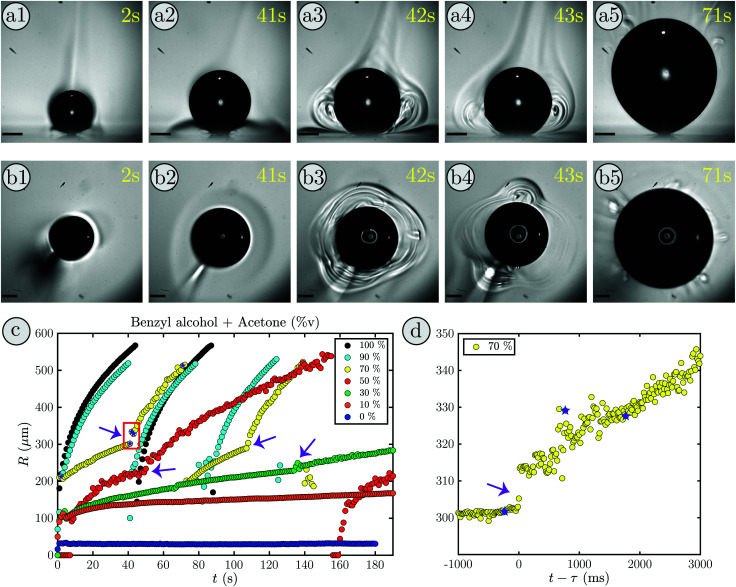
(a1)–(b5) Snapshots of a growth cycle in benzyl alcohol with an acetone ratio of *f*_A_ = 70%v, scalebar 200 μm. (a1)–(a5) show the side-view and (b1)–(b5) the bottom-view. (c) Bubble radius *R* as a function of time *t* for multiple mixing ratios of acetone and benzyl alcohol. For *f*_A_ = 70%v, 50%v and 30%v (yellow, red, green) the bubble undergoes a transition in growth behavior indicated by the purple arrows. Outside of this range (100%v, 90%v, 10%v, 0%v) no transition occurs (black, cyan, orange, blue). The snapshots (a1)–(b5) refer to the purple stars in the yellow curve (70%v) where the transition occurs in the red box. The zoom in (d) shows the radius as a function of time with respect to the transition at *τ* = 41.23 s where a jump in radius occurs. The snapshots (a2)–(a4) are marked with purple stars.

An interesting phenomena occurred when a bubble grows in a binary liquid composed of 30%v benzyl alcohol and 70%v acetone. Supplementary movie 1, ESI† shows the measured bubble radius (right) during the bubble growth viewed from the bottom (left) and the side (middle). Snapshots of the first growth cycle are shown in Fig. 1(a1)–(b5), (a) in side-view and (b) in bottom-view.

Initially, the bubble grows slowly (a1-a2), as expected, and a shadow forms close to the substrate (in Fig. 1(a2)). Similarly, in the bottom-view (in Fig. 1(b2)) the surrounding of the bubble shows a slight change in contrast. Moreover, a brighter plume above the bubble can be identified which corresponds to the bright structure in the bottom left of Fig. 1(b2). After 42 s, as shown in (a3), a sudden transition occurs and plume-like structures with rolls inside appear next to the bubble, while the shadowed region almost vanishes. The bottom-view in (b3) shows that this happens in every direction with three dominant plumes forming. Additionally, the transition is accompanied by a distinct increase in bubble size. At 43 s two plumes remain (see (b4)) and the rolls have developed further, while the depletion zone is completely gone (see (a4)). Then, the rolls and the bubble keep growing (see supplementary movie 1, ESI†) until the bubble reaches its maximum volume in (a5) and detaches. Afterwards, the whole process repeats itself. Additionally, after the transition, some bubble oscillations can be seen in supplementary movie 1 (ESI†). These oscillations will be discussed in detail in Section 4.

The contrast changes in the rolls and shadowed region only occur for mixtures and not in pure fluids. They are most pronounced if the difference in volatility and refractive index of the components of the binary liquid is large. Therefore, we interpret this as a change in the local refractive index due to a change in the local acetone concentration. Hence, the shadowed region in the beginning is considered to be a region of acetone depletion, whereas the plume above the bubble appears due to acetone enrichment caused by acetone vapor condensation. In addition, that means the rolls at the transition reflect the changes in the local acetone concentration and thereby indicate the onset of liquid motion along the rolls.

The evolution of the radius during the bubble growth is shown in Fig. 1(c) in yellow. The point at which the bubble undergoes a transition in growth mode (42 s) is clearly visible by a distinct increase in radius (red box). In movie Supplementary: Movie 2, ESI† and Fig. 1(d) the transition at *τ* = 41.23 s is shown in detail. Clearly, the jump in radius is almost instantaneous and the growth behavior changes drastically. Before the transition the growth of the radius is slow and dominated by the influx of dissolved air contained in the binary liquid as observed before for *n*-alkanes.^14^ However, after the transition the bubble grows rapidly until it detaches and the original growth mode is recovered for the new bubble. This, in conjunction with the formation of rolls around the bubble indicates that the bubble undergoes a transition from slow evaporative to convective growth. Prior studies from Zeng *et al.* (2021) support this as they have observed a strong flow near the bubble via particle image velocimetry measurements in the same system with an ethanol–water binary liquid.^18^

By systematically varying the mixing ratio of the binary liquid, the acetone fraction range in which the bubble undergoes a transition can be determined. Some exemplary cases are shown in Fig. 1(c). While the bubble undergoes a transition for intermediate acetone ratios (*f*_A_ = 30–70%v) there is none in pure acetone (black), nor in pure benzyl alcohol (blue), nor in binary mixtures close to these pure liquids (*f*_A_ = 90%v and *f*_A_ = 10%v). Moreover, with increasing *f*_A_ the bubble grows faster, irrespective of whether a transition takes place or not. This is readily explained by the fact that the bubble is mostly composed of air^11,15,17^ and the dissolved air entering the bubble during the evaporation of acetone is its main contributor. In comparison to water, where the air influx is purely diffusion driven,^11^ for the organic liquids the air solved in the fluid that enters when the fluid evaporates becomes more important. This is the case, because the solubility of air decrease in water with temperature, but increases in most organic liquids. Hence, the surrounding, heated fluid becomes slightly under-saturated in our case and hinders the growth via diffusion. Consequently, more available acetone implies that more acetone evaporates and more air enters the bubble, thereby accelerating the bubble growth.

**Fig. 2:**
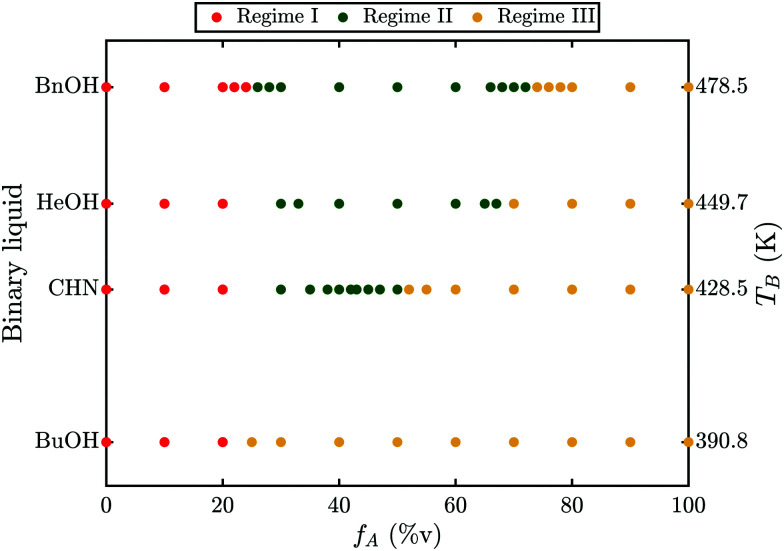
Phase diagrams of the occurrence of a transition in the growth mode as a function of the acetone ratio *f*_A_ (%v) for benzyl alcohol (BnOH), 1-heptanol (HeOH), cyclohexanone (CHN) and 1-butanol (BuOH). The respective boiling temperature of these pure fluids (*T*_B_) is shown on the right and decreases from top to bottom. The diagrams can be separated into three regimes. Regime I: no transition and slow growth occur for low acetone fractions (red). Regime II: a transition in the growth mode occurs for intermediate acetone fractions (green). Regime III: no transition as the bubble starts with fast growth immediately for high acetone fractions (yellow). The range of regime II is *f*_A_ = 26–72%v for BnOH, *f*_A_ = 30–67%v for HeOH, *f*_A_ = 30–50%v for CHN and does not occur for BuOH. For BuOH the transition from Regime I to Regime III is defined as the *f*_A_, where the largest change in growth rate occurred, because Regime II does not exist.

To investigate whether this phenomenon is limited to a specific binary liquid, the experiments have been repeated for acetone mixed with either CHN or 1-heptanol or 1-butanol. While no transition could be observed for the case of 1-butanol, both CHN and 1-heptanol exhibit a transition and a similar behavior to benzyl alcohol. The results for these binary liquids are summarized in a phase diagram in Fig. 2. For all combinations shown, three different regimes can be identified. First, for low acetone ratios the bubble grows slowly and undergoes no transition (regime I, red). Then, for intermediate ratios the bubble undergoes a transition in growth mode (regime II, green). Finally, for high acetone ratios no transition occurs and the bubble grows fast and detaches (regime III, yellow). Regime II ranges from *f*_A_ = 26–72%v for BnOH (a), *f*_A_ = 30–67%v for 1-heptanol (b), *f*_A_ = 30–50%v for CHN (c) and does not exist for 1-butanol. Clearly, the size of regime II depends on the fluid as it decreases from BnOH via 1-heptanol, and CHN to 1-butanol. Comparing the liquids properties, listed in Table 1, the boiling temperature (*T*_B_, right axis in Fig. 2) matches the decrease in regime II and hence we identify *T*_B_ as the most probable determining parameter. In order to understand why *T*_B_ should be relevant and what mechanism causes the bubble to undergo a transition, a more detailed examination of this phenomenon is required.

### 3.2 Behavior at the transition

**Fig. 3:**
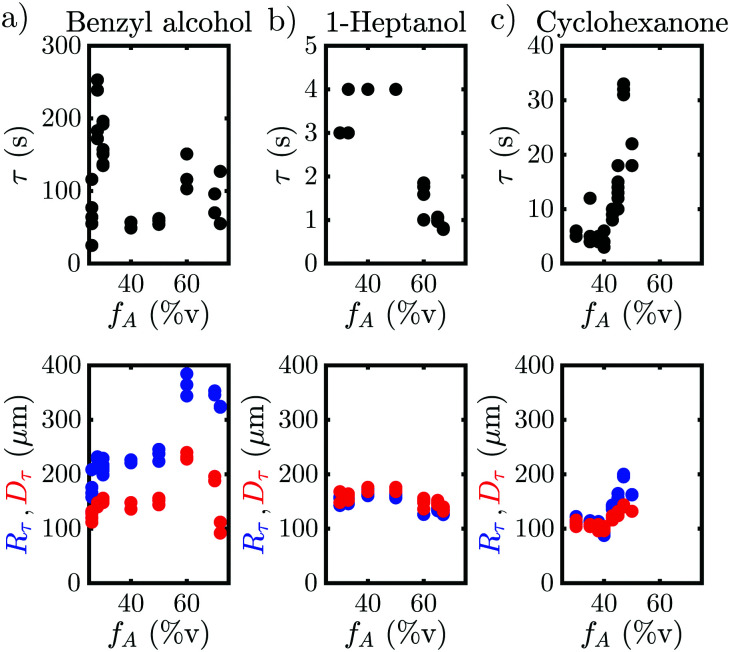
Time *τ*, bubble radius *R*_*τ*_ (blue) and footprint diameter *D*_*τ*_ (red) at the transition of the growth mode as a function of the acetone fraction *f*_A_ (%v) for benzyl alcohol (a), 1-heptanol (b) and cyclohexanone (c).

**Fig. 4:**
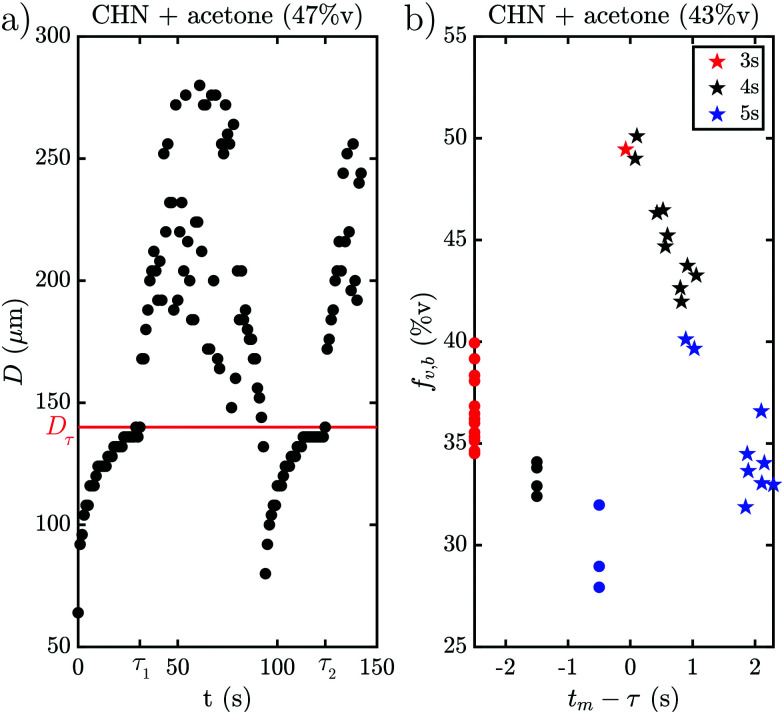
Footprint diameter *D* as a function of time for cyclohexanone with *f*_A_ = 47%v (a). Transitions occur at *τ*_1_ = 31 s and *τ*_2_ = 124 s with *D*_*τ*_ = 140 μm. *D* (*t*) stagnates before the transition and then increases afterwards. Vapor fraction of the bubble volume *f*_v,b_ (b) for cyclohexanone with *f*_A_ = 43%v as a function of the time the measurement took place *t*_m_ with respect to the transition at *τ*. Different colors denote different laser pulse lengths. Right after the transition *f*_v,b_ (*t*) jumps from approx. 30%v to about 50%v and then decreases within a few seconds to the level before the transition although the bubble has changed its growth mode.

The behavior when the bubble undergoes a transition in growth mode is shown in Fig. 3 for the three cases BnOH (a), 1-heptanol (b) and CHN (c). The top row shows the time *τ* after nucleation at which the transition occurs and the bottom row the footprint diameter *D*_*τ*_ (red) and the bubble radius *R*_*τ*_ (blue) right before the transition as a function of the acetone ratio *f*_A_. Noticeably, *τ* varies drastically between the different fluids from a rapid transition *τ* < 5 s for 1-heptanol via multiple seconds *τ* < 40 s for CHN up to a few minutes *τ* < 260 s for BnOH. Moreover, we note that *τ* does not follow the trend in *T*_B_ as *T*_B_ (HeOH) > *T*_B_ (CHN), but instead seems to depend on the surface tension (*σ*), as *σ* (BnOH) > *σ* (CHN) > *σ* (HeOH). On the other hand, *R*_*τ*_ is of the same order of magnitude for all three binary liquids ranging from *R*_*τ*_ = 158–385 μm for BnOH, *R*_*τ*_ = 126–183 μm for 1-heptanol and *R*_*τ*_ = 84–200 μm for CHN. Surprisingly, there is a sudden increase in *R*_*τ*_ and *D*_*τ*_ for the case of BnOH *f*_A_ = 60%v. Remarkably, *D*_*τ*_ lies almost completely between 100–200 μm for all measured cases (except for the aforementioned BnOH with *f*_A_ = 60%v) and remains almost constant. Considering that the bubble cross section *D*_*τ*_ at the wall at transition is rather constant, while *τ* and *R*_*τ*_ vary for the various cases, it seems worthwhile to further investigate the region close to the wall and the exact temporal behavior of the footprint diameter *D* (*t*). An exemplary case of the whole evolution of the footprint diameter *D* (*t*) is shown in Fig. 4(a) for CHN with *f*_A_ = 47%v. While *D* (*t*) initially grows, it starts to stabilize before each transition with *D*_*τ*_ = 140 μm at *τ*_1_ = 31 s and *τ*_2_ = 124 s, respectively. Right after each transition at *τ*, *D* (*t*) increases rapidly. While this is similar to the behavior of the bubble radius *R*(*t*), it differs by the stabilization before the transition and the fact that *D*_*τ*_ is comparable for all binary liquids. Furthermore, if we take another look at supplementary movie 2 (ESI†), the gradients in grey-scale and the plumes appear first at the three-phase contact line. All this indicates that the mechanism originates at the three-phase contact line.

Another question which arises is whether the drastic change in bubble volume at the transition is caused by an influx of gas, evaporation or a combination of both? To find out, the bubble composition has been determined by studying the shrinkage of the bubble after exposure to a laser pulse, as done by Zaytsev *et al.*^16^ The method employed in that paper is based on the fact that the condensation of vapor is orders of magnitude faster than the dissolution of gas. Hence, the initial drop in bubble volume during its shrinkage reflects the vapor fraction. The remainder of the bubble content is gas (*i.e.* air). The result of this method is shown in Fig. 4(b) for CHN with *f*_A_ = 43%v, where red, black and blue indicate a laser pulse length of 3 s, 4 s and 5 s, respectively. Without a transition the bubble consists mostly of gas with a vapor fraction *f*_v,b_ ≈ 33%v, which is slightly lower for longer exposure (blue curve) as the bubble has more time to grow via diffusion. However, right after the transition the vapor fraction instantly jumps to *f*_v,b_ = 50%v and then decreases within 2 s back to the pre-transition level of *f*_v,b_ ≈ 33%v. So, the transition is accompanied by a drastic change in composition and the mechanism thus explains the initial burst of vapor production and bubble growth. With this we have collected enough hints to explain the mechanism.

### 3.3 Driving mechanism of the transition

Summarizing the earlier results, the boiling temperature of the fluid determines whether a transition takes place. The evaporation happens at the stagnating three-phase contact line and is accompanied by an initial burst of vapor production. Combining all these facts we propose a mechanism that explains why the bubble undergoes a transition in the growth mode. First, we determine the Jakob number Ja, which is the ratio between the heat needed to reach the boiling temperature of the liquid (sensible heat) and the latent heat of vaporization. For all cases that exhibit a transition we find 0.2 < Ja < 0.7, showing that most energy is spend on the evaporation of the liquid and not on heating it. Consequently, evaporative cooling is important in our system.

**Fig. 5:**
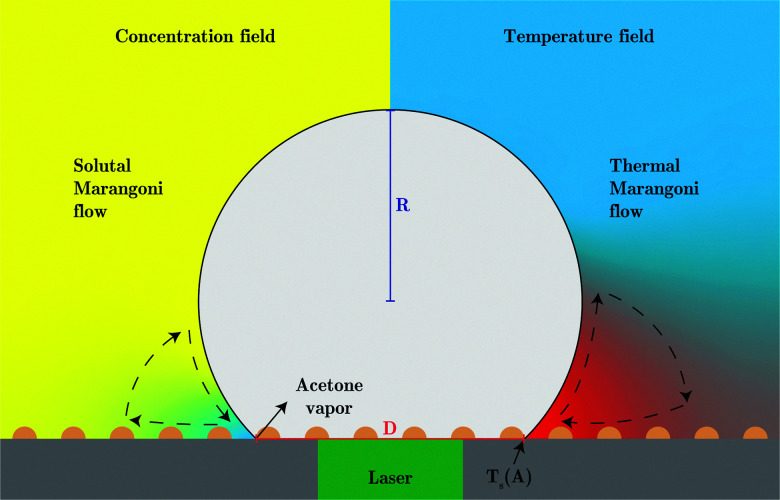
Sketch of the proposed mechanism that causes the transition in the growth mode (not to scale). Left: Concentration field with yellow and blue denoting high and low acetone concentration respectively. A strong solutal Marangoni flow towards the three-phase contact line occurs as a strong surface tension gradient builds up due to acetone evaporation into the bubble. Right: Temperature field with red and blue denoting high and low temperatures, respectively. As the surface tension decreases with increasing temperature the much weaker thermal Marangoni flow points away from the three-phase contact line which at the transition in growth mode is at the spinodal temperature of acetone (*T*_s_ (*A*) = 457 K). Additionally, the footprint diameter *D* (red) and bubble radius *R* (blue) are sketched in the schematic.

Initially, the bubble grows fast and exceeds the area that is heated by the laser. Then, the bubble growth slows down and the footprint diameter *D* (*t*) remains constant. As *D* (*t*) stagnates, acetone replenishment only occurs via diffusion and hence the local acetone concentration decreases, thus reducing the evaporation rate. Since the evaporative cooling decreases, while the heat influx due to the continuous laser irradiation remains the same, the temperature at the contact line slowly increases. As long as the boiling temperature of the less volatile fluid is comparable to the spinodal temperature of acetone *T*_s_ (*A*) ≈ 0.9*T*_c_ = 457 K,^35^ it should not evaporate significantly. Thereby, it neither limits the temperature increase due to latent heat, nor contributes to the vapor fraction *f*_v,b_ in the bubble. Once the temperature of the contact line reaches *T*_s_ (*A*), the latent heat of vaporization of acetone vanishes and almost all the remaining acetone in the mixture near the contact line will instantly evaporate without energy cost. This leads to a jump in bubble radius *R* (*t*) and also increases the vapor fraction *f*_v,b_ inside the bubble as the additional volume is solely composed of vapor. At the same time, the rapid evaporation causes a sudden decrease in the local acetone concentration *f*_A,l_ at the contact line. Hence, a strong gradient in surface tension to the ambient *f*_A_ is created along the bubble, as *f*_A,l_ ≪ *f*_A_, which leads to Marangoni flow. This flow ambient liquid along the bubble interface and marks the onset of streaming, as sketched in Fig. 5. The thermal Marangoni flow can be neglected, as the Marangoni numbers for the solutal and thermal Marangoni flow are 
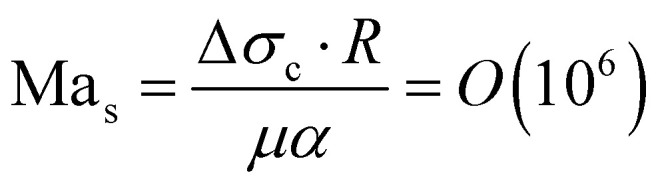
 and 
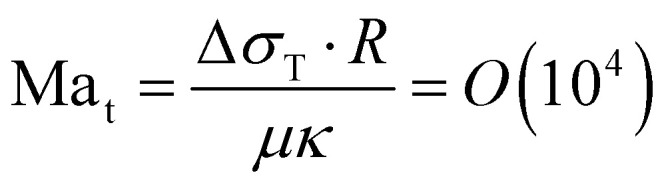
, *i.e.* Ma_s_ ≫ Ma_t_. Here *μ* is the dynamic viscosity of the binary liquid, Δ*σ*_c_ and Δ*σ*_T_ are the surface tension gradients caused by concentration and temperature and *α* and *κ* are the mass and thermal diffusivity. Once the flow sets in, it will supply fresh fluid with the ambient acetone concentration *f*_A_ to the contact line, where acetone will preferentially evaporate. Consequently, a concentration gradient is maintained, allowing the streaming to persist. Besides that, the incoming fresh fluid will reduce the size of the depletion zone. As acetone contains more dissolved air than the less volatile fluids and evaporates easier, the growth of the bubble will accelerate once convection sets in and be similar to pure acetone (as observed in Fig. 1). As the growth is again dominated by the influx of dissolved air, the bubble composition shifts back towards the lower vapor fraction shortly after the transition.

Because this mechanism predicts a transition only when the boiling temperature *T*_b_ of the less volatile fluid is comparable to the spinodal temperature *T*_s_ (*A*) of the volatile one, it explains why no transition occurs for 1-butanol and why regime II increases with *T*_b_. Besides this, it also explains why no transition occurs close to the pure fluids. For high *f*_A_ the bubble grows too fast to reach *T*_s_ (*A*) at the contact line. On the other hand, for low *f*_A_ the concentration gradient to the local *f*_A,l_ is small and therefore the Marangoni flow becomes weak and not enough acetone can be resupplied to enhance the growth. Besides this, it provides a potential explanation to the surprising behavior of *τ* that seems to depend on the surface tension. It might be caused by the contact angle of the fluid, as it increases on the hydrophilic substrate from BnOH via CHN to HeOH, presumably due to their surface tensions. As the contact angle increases and approaches 90°, the heated liquid layer trapped between the bubble and the substrate shrinks and thereby reduces the distance between *f*_A,l_ and *f*_A_. In turn, this could facilitate the onset of the solutal Marangoni flow and thereby decrease *τ*.

**Fig. 6:**
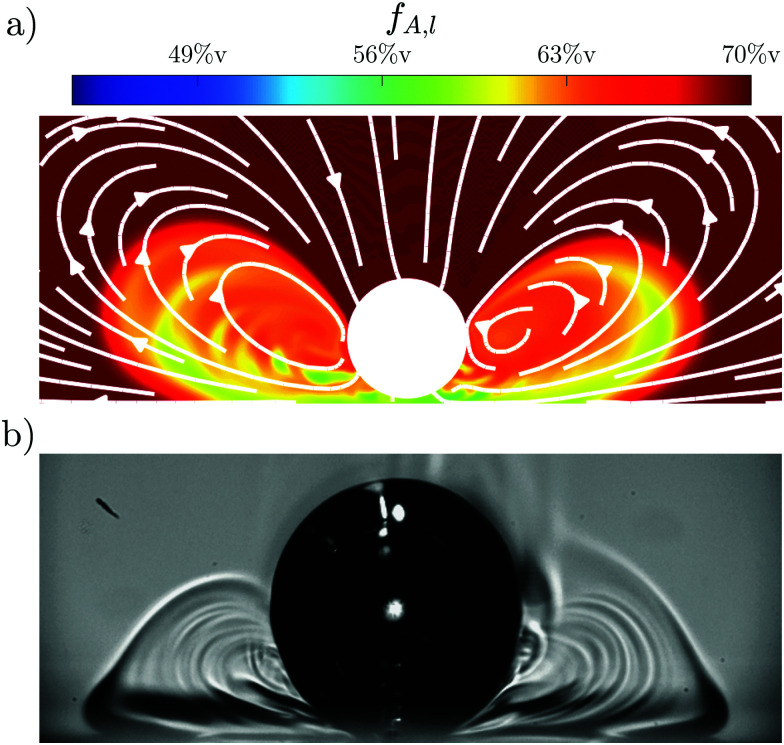
(a) Simulation of the Marangoni flow at a catalytic plane for Ma_s_ = 5 × 10^5^, white arrows indicate the flow inside the fluid. The local acetone concentration *f* is shown as a fraction of the ambient *f*_A_ = 70%v, with plumes of lower *f* originating at the interface below the bubble. (b) Experimental data for Ma_s_ = 1.2 × 10^6^. Different shades of grey indicate concentration gradients. Both simulation and the experiment are a*t t*–*τ* = 0.5 s after the transition, with *R*_*τ*_ = 300 μm. However, the bubble in the experiment keeps growing and therefore is 5% larger than in (a). The shown and affected region is significantly larger in the simulation, however, there is good qualitative agreement of the structures in the concentration field between simulation and experiment.

In order to verify this suggested mechanism, simulations for a Marangoni instability at a catalytic plane are performed (details of this simulation can be found in Li *et al.* (2021)^19^). The result of the simulations for Ma_s_ = 5 × 10^5^ is shown in Fig. 6(a) and compared to the experimental data. Both cases show the bubble at *t*–*τ* = 0.5 s with *R*_*τ*_ = 300 μm. However, the simulations do not take into account bubble growth, so that the bubble is 5% larger in the experiment compared to the simulation with *R*_e_ (*t*) = 315 μm and *R*_s_ (*t*) = 300 μm, respectively. For the simulations the local acetone concentration *f*_A,l_ is shown via a color map, while the resulting flow is indicated by white arrows. The concentration gradients in the experimental data are indicated by different shades of gray. There is good qualitative agreement between the simulations and experiments as both show the same rolls and general structure. This further supports that the mechanism originates from a Marangoni instability.

The mechanism also predicts the same transition for other binary liquids as long as *T*_b_ of the less volatile fluid is comparable to *T*_s_ of the volatile one. One such combination is BnOH (*T*_b_ = 478.5 K) with ethanol (*T*_s_ (*E*) ≈ 464 K).^35^ Indeed, a transition could also be observed for this binary fluid as shown in supplementary movie 3 (ESI†) though no systematic analysis has been performed.

Summarizing this section, we conclude that the proposed mechanism is consistent with all our observations and explains why and when a bubble undergoes a transition in growth behavior.

## 4 Bubble oscillations

As eluded in Section 3, the bubble not only grows faster after the transition, but can also be accompanied by oscillations. Two different kinds of motion can be observed: the bubble can either oscillate about its spherical shape or be displaced partially from the laser spot and orbit around it. While both clockwise and counterclockwise orbiting can occur, once one is present the direction will not change anymore during the bubble growth. Supplementary movie 4 (ESI†) for 1-hepanol with *f*_A_ = 77%v and supplementary movie 5 (ESI†) with *f*_A_ = 72%v respectively show exemplary cases of these motions in bottom- and side-view.

**Fig. 7:**
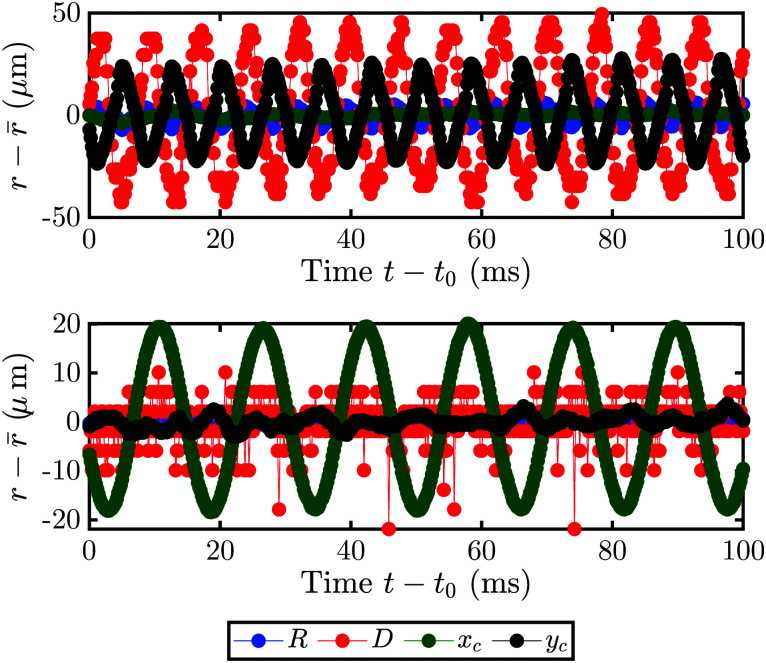
Radius *R* (blue), footprint diameter *D* (red) and bubble center (*x*_c_ green, *y*_c_ black) motion as a function of time for two different cases. *x* is parallel and *y* perpendicular to the substrate. Oscillations in those directions correspond to a rotation around the laser spot and an elongation of the bubble, respectively. For 1-heptanol with *f*_A_ = 70%v the footprint diameter *D* and the *y*_c_ motion oscillate (a), whereas for *f*_A_ = 72%v the bubble only oscillates along the substrate in *x*_c_. Both cases are for a laser power of 140 mW and taken at *t*_0_ = 7 s of continued exposure.

In order to understand this phenomenon, we investigated the behavior on the much shorter timescale of milliseconds. The motion of the bubble is recorded in side-view and parameters such as the motion of its center-coordinates *x*_c_ (*t*), *y*_c_ (*t*), radius *R* (*t*) and the footprint diameter *D* (*t*) are extracted. Fig. 7 shows the bubble motion as deviation of the mean for an example of the oscillation (a) and the orbiting (b) from *t*_0_ = 7 s. The directions *x* and *y* are parallel and perpendicular to the substrate, respectively. In the case of an oscillating bubble, the bubble remains pinned to the laser spot (as *x*_c_ (*t*) remains constant) and oscillates in shape as *y*_c_ (*t*) shows a clear periodic signal. Interestingly, *D* (*t*) oscillates with the same frequency as *y*_c_ (*t*), albeit with a phase shift of about 180°. In contrast, the orbiting motion is not accompanied by any other motion as only *x*_c_ (*t*) oscillates. Additionally, the frequency of the oscillation and orbiting are clearly different. However, a bubble can switch from one kind of motion to another during its growth.

**Fig. 8:**
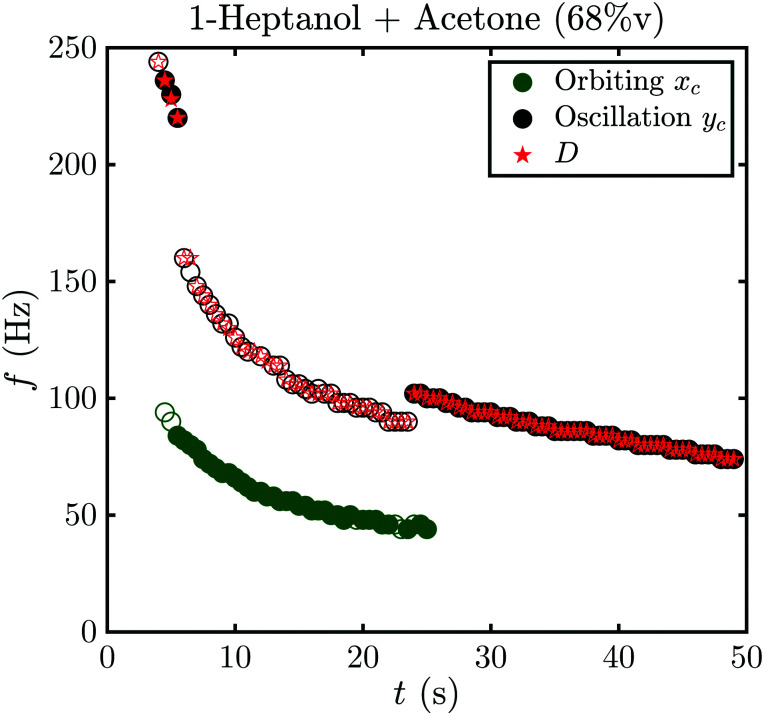
Frequency *f* of the oscillation of the coordinates of the bubble center *y*_c_ (black), *x*_c_ (green), and the footprint diameter *D* (red) as a function of time *t* during the growth of a bubble in 1-heptanol with *f*_A_ = 68%v and a laser power of 100 mW. Full and open symbols represent a large and small amplitude of the oscillation, respectively. The dominant motion changes at *t* = 5.5 s and *t* = 24 s. *D* and *y*_c_ have the same frequency throughout and the frequency of *y*_c_ when the amplitude is small is twice the frequency of *x*_c_.

An example of this is shown in Fig. 8 for *f*_A_ = 68%v, where the frequency during the whole life of the bubble is shown as a function of time. Closed symbols represent the main motion of the bubble, whereas open symbols also consider small amplitude oscillations. In both cases the bubble starts with shape oscillations *y*_c_ (*t*) (black) which are accompanied by oscillations of the footprint diameter *D* (*t*) (red). Then, the motion switches to orbiting *x*_c_ (*t*) (green) with a brief moment at *t* = 5.5 s where both motions are present and overlap. If only large amplitude motions are considered, there is no other motion present until shape oscillations appear again at *t* = 24 s and become dominant at *t* = 25 s. However, if small amplitude oscillations are not neglected oscillations in *y*_c_ (*t*) and *D* (*t*) become visible during the orbiting. Surprisingly, those oscillations have about twice the frequency of *x*_c_ (*t*) with 
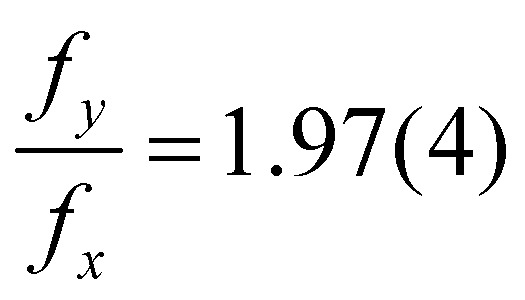
. Additionally, they have a different frequency compared to the same motion with a large amplitude (closed symbols), as clearly visible at *t* = 24 s. Therefore, in the following analysis only the large amplitude oscillations will be considered. The reason why the bubble switches motion is not clear, however, one possible explanation could be that it is caused by an asymmetry. *E.g.*, the local pinning could vary and lead to a small displacement of the bubble center from the laser spot. Then, the bubble would first move along a line and then undergo a transition into an orbiting motion. We have indeed observed such a behavior, though no detailed analysis has been performed.

**Fig. 9:**
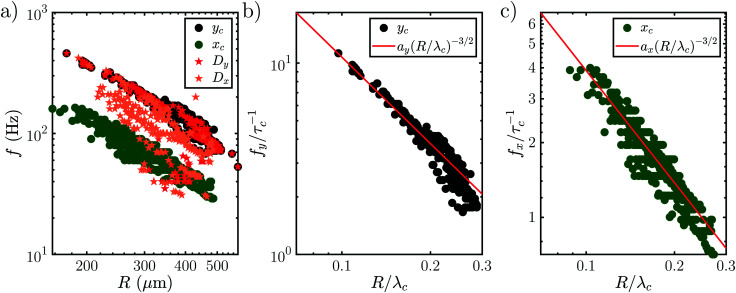
Data of all oscillation measurements combined. (a) Oscillation frequency *f* as a function of the bubble radius *R*. *D*_*y*_ (red) and *D*_*x*_ (orange) denote the frequency of the footprint oscillation when shape oscillations or orbiting are dominant respectively. The frequencies match those for shape oscillations, but do not for the orbiting motion. Both frequencies are fitted with a power law *f*_i_/*τ*_c_^−1^ = *a*_i_·(*R*/*λ*_c_)^−3/2^ (red), see (b) and (c). The prefactors are *a*_*y*_ = 0.339(3) for the shape oscillations and *a*_*x*_ = 0.124(2) for the orbiting motion.

Interestingly, all data for the oscillation and orbiting modes at different laser powers and concentration collapse on one curve, as shown in Fig. 9(a). Stars show the motion in *D* (*t*) if an oscillation (*D*_*y*_ (*t*)) or orbiting (*D*_*x*_ (*t*)) is detected. Similar to Fig. 7, the frequency of *D* (*t*) matches the oscillation, but not the orbiting. However, clearly both kinds of motion follow a power law with 
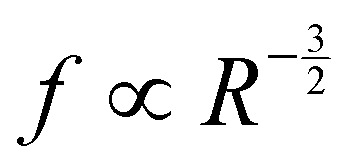
.

In order to understand this behavior we have another look at Fig. 5 where the situation is similar. In this case the bubble has grown rapidly and hence the temperature at the contact line is lower. Nevertheless, both thermal and solutal Marangoni flows are still present, since gradients in temperature and concentration remain (as sketched in Fig. 5). Similar to Section 3.3 it is reasonable to neglect the effect of the thermal Marangoni flow at the interface because Ma_s_ ≫ Ma_t_. Then, the solutal Marangoni flow pushes the contact line inwards and in response the bubble elongates perpendicular to the substrate (along the *y*-axis). However, surface tension acts against this deformation and drives the bubble back to its spherical shape. Consequently, the contact line extends again and the whole process repeats itself, as for a spring-mass system. Basically, we have a small perturbation driven by solutal Marangoni flow and surface tension acts against it. Lamb determined the response frequency *ω* of a gas bubble in another inviscid fluid to a small perturbation of the interface using surface waves as^36,37^
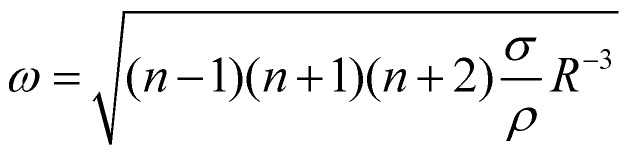
where *n* is the mode number, *R* the bubble radius, *σ* the surface tension, and *ρ* the density of the surrounding fluid, which provides the inertia of the oscillation. Using the most important mode of vibration *n* = 2, the scaling law 
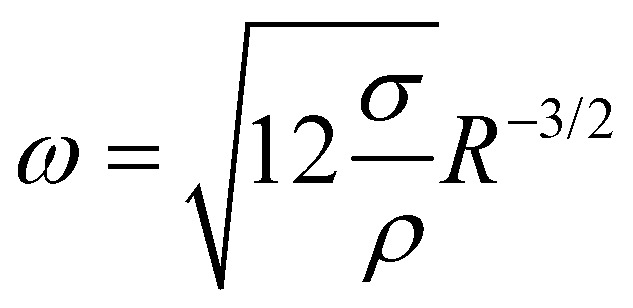
 follows. If also viscosity is taken into account, the angular frequency *ω* remains the same, though damping occurs with 
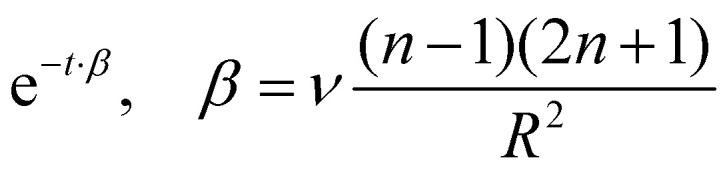
, where *ν* is the kinematic viscosity of the surrounding fluid.^36,37^ This predicted power law matches the experimental findings very well. We non-dimensionalize *R* and *f* with the capillary length 
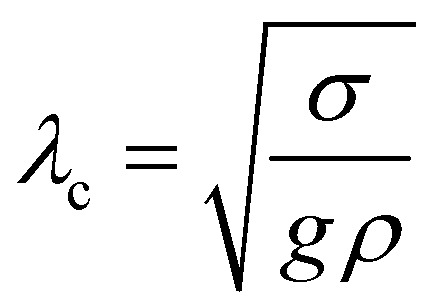
 and the characteristic time 
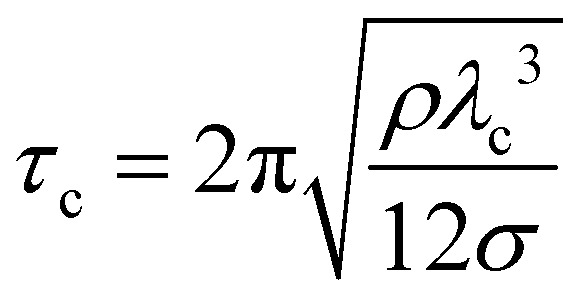
, with *g* the gravitational acceleration. Both motions are shown in Fig. 9(b) and (c) with a fitted power law *f*_i_*τ*_c_ = *a*_i_ (*R*/*λ*_c_)^−3/2^, *i* = *x*, *y* with prefactors *a*_i_. We find *a*_*x*_ = 0.124(2) and *a*_*y*_ = 0.339(3) which are in order of magnitude agreement with the theoretical prediction (*a*_*y*_ = 1). The comparatively lower prefactors are reasonable, as the theoretical prediction is for a free bubble, whereas in our experiments the bubble is at a wall. Analogous to *e.g.* the narcissus effect in acoustically driven shape oscillations, the wall can be replaced by a mirror image of the bubble. Both experiments and theory show that this would reduce the resonance frequency and can be understood as effectively increasing the added mass and the size of the oscillating bubble.^38,39^

Surprisingly, the orbiting motion also obeys the power law derived for shape oscillations. In order to understand this we have to take into account the previously neglected small amplitude oscillations (see Fig. 8). As their frequency is the second harmonic of the orbiting frequency and varies from the large amplitude oscillations, we assume they couple with the orbiting motion. We assume that they couple to the second harmonic of the dominant orbital motion, because it is the closest harmonic to their original frequency when shape oscillations are dominant. The small shape oscillations can be explained by the same mechanism used for the large amplitude oscillations, though the prefactor may change due to the coupling with the orbiting. Because of the coupling, the scaling of the small shape oscillation should transfer to the orbiting motion, too. Hence, it follows the same behavior as the large amplitude shape oscillations.

## 5 Conclusions and outlook

We experimentally found the new phenomenon that a transition in growth behavior of plasmonic bubbles occurs for binary liquids. This transition is driven by solutal Marangoni flow and massive evaporation once the spinodal temperature (*T*_s_) of the more volatile fluid has reached the three-phase contact line. Consequently, this transition can only be observed in binary fluids, where the boiling temperature of the less volatile fluid (*T*_b_) is comparable to *T*_s_. The Marangoni flow also causes the bubble to deform. This deformation is opposed by surface tension, leading to bubble oscillations. Both shape oscillations and an orbiting motion around the laser spot are found and they follow the same power law 
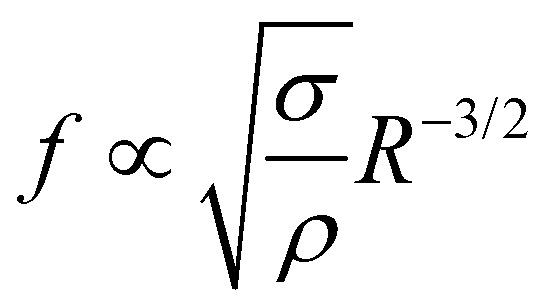
, matching the expectation for shape oscillations. Surprisingly, the orbiting motion also follows the same power law, because it is coupled to small amplitude shape oscillations that persist during the orbiting.

Both the transition and the oscillations cause motion in a volume more than an order of magnitude larger than the bubble and hence can be very beneficial for mixing, *e.g.* in catalysis or microfluidics. This is particularly relevant, because a wide range of fluids can be used as long as they satisfy that one is significantly more volatile (*i.e. T*_b_ ≲ *T*_s_).

Further experiments that study the effect of other parameters, *e.g.* viscosity via silicon oils, are suggested. Another interesting aspect of the mechanism is that it does not require plasmonic heating, but only a localized heat source. Thus, we envision that our results could be reproduced with other heat sources, such as small resistive heaters, which removes the need of optical access to benefit from the enhanced mixing.

## Author contributions

The experiments have been performed by M. Detert and the simulations by Y. Chen. The experiments were conceptualized by M. Detert, H. J. W. Zandvliet and D. Lohse. M. Detert, H. J. W. Zandvliet and D. Lohse have contributed to the interpretation of the data and to writing the manuscript.

## Conflicts of interest

There are no conflicts to declare.

## Supplementary Material

SM-018-D2SM00315E-s001

SM-018-D2SM00315E-s002

SM-018-D2SM00315E-s003

SM-018-D2SM00315E-s004

SM-018-D2SM00315E-s005
